# Effect of Remote Ischaemic Conditioning in Oncology Patients Undergoing Chemotherapy: Rationale and Design of the ERIC‐ONC Study—A Single‐Center, Blinded, Randomized Controlled Trial

**DOI:** 10.1002/clc.22507

**Published:** 2016-01-25

**Authors:** Robin Chung, Angshuman Maulik, Ashraf Hamarneh, Daniel Hochhauser, Derek J. Hausenloy, J. Malcolm Walker, Derek M. Yellon

**Affiliations:** ^1^ The Hatter Cardiovascular Institute University College London London United Kingdom; ^2^ Research Department of Oncology The Cancer Institute, University College London London United Kingdom; ^3^ Cardiovascular and Metabolic Disorders Program Duke University–National University of Singapore Medical School Singapore

## Abstract

Cancer survival continues to improve, and thus cardiovascular consequences of chemotherapy are increasingly important determinants of long‐term morbidity and mortality. Conventional strategies to protect the heart from chemotherapy have important hemodynamic or myelosuppressive side effects. Remote ischemic conditioning (RIC) using intermittent limb ischemia‐reperfusion reduces myocardial injury in the setting of percutaneous coronary intervention. Anthracycline cardiotoxicity and ischemia‐reperfusion injury share common biochemical pathways in cardiomyocytes. The potential for RIC as a novel treatment to reduce subclinical myocyte injury in chemotherapy has never been explored and will be investigated in the Effect of Remote Ischaemic Conditioning in Oncology (ERIC‐ONC) trial (clinicaltrials.gov NCT 02471885). The ERIC‐ONC trial is a single‐center, blinded, randomized, sham‐controlled study. We aim to recruit 128 adult oncology patients undergoing anthracycline‐based chemotherapy treatment, randomized in a 1:1 ratio into 2 groups: (1) sham procedure or (2) RIC, comprising 4, 5‐minute cycles of upper arm blood pressure cuff inflations and deflations, immediately before each cycle of chemotherapy. The primary outcome measure, defining cardiac injury, will be high‐sensitivity troponin‐T over 6 cycles of chemotherapy and 12 months follow‐up. Secondary outcome measures will include clinical, electrical, structural, and biochemical endpoints comprising major adverse cardiovascular clinical events, incidence of cardiac arrhythmia over 14 days at cycle 5/6, echocardiographic ventricular function, N‐terminal pro‐brain natriuretic peptide levels at 3 months follow‐up, and changes in mitochondrial DNA, micro‐RNA, and proteomics after chemotherapy. The ERIC‐ONC trial will determine the efficacy of RIC as a novel, noninvasive, nonpharmacological, low‐cost cardioprotectant in cancer patients undergoing anthracycline‐based chemotherapy.

## Introduction

Cancer affects more than 1 in 3 people in their lifetime and, together with cardiovascular diseases, remains the leading causes of morbidity and mortality in developed nations. Cancer outcomes continue to improve such that long‐term 10‐year survival for all cancers now stands at 50%, and 80% or better for breast, prostate, Hodgkin's lymphoma, and melanoma.^1^ Cancer survivors will make up more than 5% of the US population by 2022,^2^ and nearly 25% of the UK population aged 65 years and older by 2040.^3^ These welcome improvements in primary cancer outcomes give rise to a substantial survivor population at risk of long‐term cardiovascular consequences, either due to the cancer treatment itself or due to traditional cardiovascular risks. Cardiovascular mortality in cancer patients is increased due to adverse coronary outcomes,^4^ and one‐third of long‐term cancer survivors will die from cardiovascular causes.^5^

Even in the era of targeted cancer treatments, anthracyclines, either alone or in combination, remain the mainstay for many types of cancer treatments including breast, lymphoma, sarcoma, and leukemia, but are almost invariably limited by systemic and cardiac side effects. Thus, cancer cardioprotection is an important aspect of long‐term patient care.

## Cardiotoxicity of Anthracyclines

Anthracyclines exert their cytotoxic effect via topoisomerase II inhibition and intercalation of DNA.^6^ However, this effective—but nonselective—antitumor mechanism has cardiotoxic consequences. Anthracycline cardiotoxicity is classically attributed to lipid peroxidation and iron‐complex reactive oxidation species (ROS),^7^, ^8^, ^9^, ^10^ to which the myocyte is particularly vulnerable owing to a lack of catalase, glutathione peroxidase, and limited regenerative capacity.^11^, ^12^, ^13^ Cardiomyocytes are vulnerable to anthracycline injury because they express topoisomerase II‐β,^10^, ^14^ but additional myocyte damage is caused through calcium dysregulation and mitochondrial permeability transition pore (MPTP) disruption.^15^, ^16^, ^17^

Anthracycline cardiotoxicity, defined initially as clinical heart failure, was reported soon after the anthracyclines' widespread introduction in the 1960s.^18^ This led to the current practice of limiting the cumulative doxorubicin dose to a lower cutoff of 400 mg/m^2^. Longitudinal studies documented high mortality from anthracycline‐induced congestive heart failure—60% at 2 years^18^, ^19^, ^20^—with image‐based subclinical cardiac dysfunction occurring in at least 9%^21^ to 18%.^22^

## Cardioprotection

**Figure 1:**
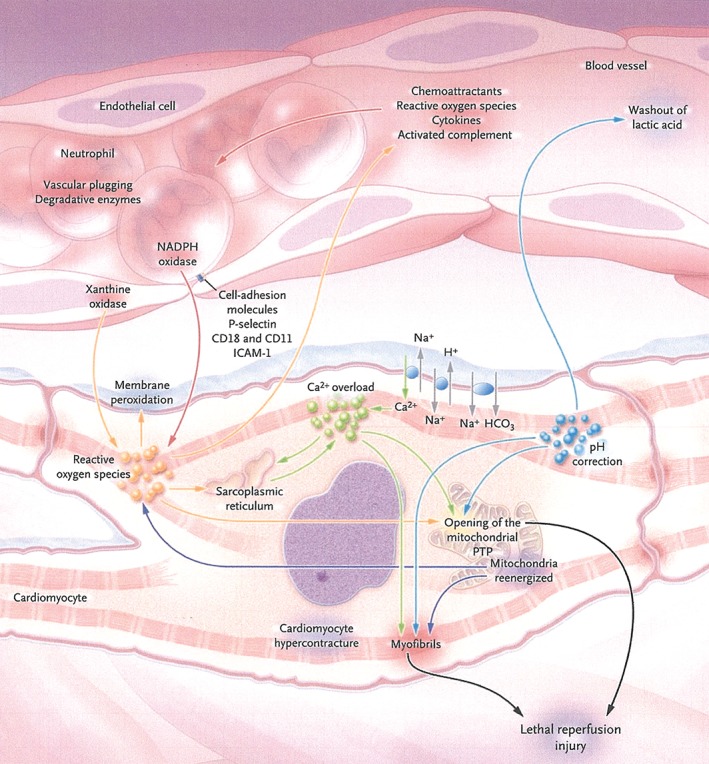
Reperfusion injury and doxorubicin cardiotoxicity pathways. Pathological activation of RoS formation, calcium overload, and altered mitochondrial respiration in reperfusion injury are also found in anthracycline cardiotoxicity. (Reproduced under license from Yellon and Hausenloy^86^ and the Massachusetts Medical Society/New England Journal of Medicine.). Abbreviations: ICAM‐1, intercellular cell adhesion molecule‐1; NADPH, nicotinamide adenine dinucleotide phosphate hydrogen; PTP, permeability transition pore.

Cardioprotection to limit myocardial damage from anthracycline chemotherapy has to date focused on 2 approaches: modified doxorubicin preparations and pharmacological treatments. Revised infusion strategies using lower dose,^23^ continuous infusion,^24^ liposomal doxorubicin,^25^, ^26^ iron chelation,^27^, ^28^, ^29^ and anti–heart failure medications have all demonstrated some success in mitigating myocardial injury. Up to half of myocyte cell death during acute myocardial infarction is caused by reperfusion itself. Murry et al described ischemic conditioning as endogenous activation of cardioprotective mechanisms to limit myocardial infarct size through repeated cycles of ischemia‐reperfusion.^30^ Anthracycline cardiotoxicity and reperfusion cause myocyte injury via common signaling pathways and molecular targets (Figure 1). Liposomal peroxidation, reactive oxidation species, calcium overload,^31^, ^32^, ^33^ and mitochondrial respiration/gene expression^17^, ^34^, ^35^, ^36^, ^37^ are affected in both injury processes. Thus, remote ischemic preconditioning, cardioprotective in ischemic injury, may also confer cardioprotection from anthracycline chemotherapy.

## The Effect of Remote Ischaemic Conditioning in Oncology Patients Study

### Hypothesis

Remote ischemic conditioning (RIC), via repeated cycles of inflation and deflation of a peripheral arm blood pressure cuff, reduces myocardial injury in anthracycline‐treated cancer patients.

### Study Objectives

The Effect of Remote Ischaemic Conditioning in Oncology Patients (ERIC‐ONC) study will be a proof‐of‐concept study to investigate the effect of RIC in oncology patients. The study objective is to determine whether RIC reduces cardiac biomarker evidence of subclinical myocardial cardiotoxicity during anthracycline‐based chemotherapy treatment. Primary and secondary outcome measures are detailed in the Study Endpoints section and summarized in Table 1.

**Table 1:** Study Outcome Measures

Outcome	Endpoint	Time Frame
Primary outcome	High‐sensitivity troponin‐T	Baseline, 6–24 hours after the end of each chemotherapy infusion, 1, 3, 6, 12 months follow‐up
Secondary outcomes	Major adverse clinical cardiovascular event	1, 3, 6, 12 months follow‐up
	Myocardial infarction	
	Clinical heart failure requiring admission	
	Life‐threatening arrhythmia (ventricular tachycardia, ventricular fibrillation)	
	Atrioventricular block requiring pacemaker	
	Cardiac or cancer death	
	Echocardiographic longitudinal function	Baseline, 3, 12 months follow‐up
	Global longitudinal strain (%)	
	Incidence of cardiac arrhythmia	Zio XT ambulatory ECG patch worn at start of chemotherapy cycle 5 of 6 (penultimate cycle) for 2 weeks continuous monitoring
	Atrial fibrillation/flutter	
	Supraventricular tachycardia (AVNRT)	
	Ventricular tachycardia	
	Atrioventricular block	
	NT pro‐BNP level	Baseline, 3 months follow‐up
	MicroRNA^a^	Baseline, 3 months
	Mitochondrial DNA^a^	
	Urine proteomics and protein expression markers^a^	

Abbreviations: AVNRT, atrioventricular nodal reentrant tachycardia; ECG, electrocardiogram; NT pro‐BNP, N‐terminal pro‐brain natriuretic peptide.

a Metabolic markers for microRNA, mitochondrial DNA, and urine proteomics will be collected in 20 participants (control, n = 10; remote ischemic conditioning, n = 10).

## Methods

### Study Design

**Figure 2:**
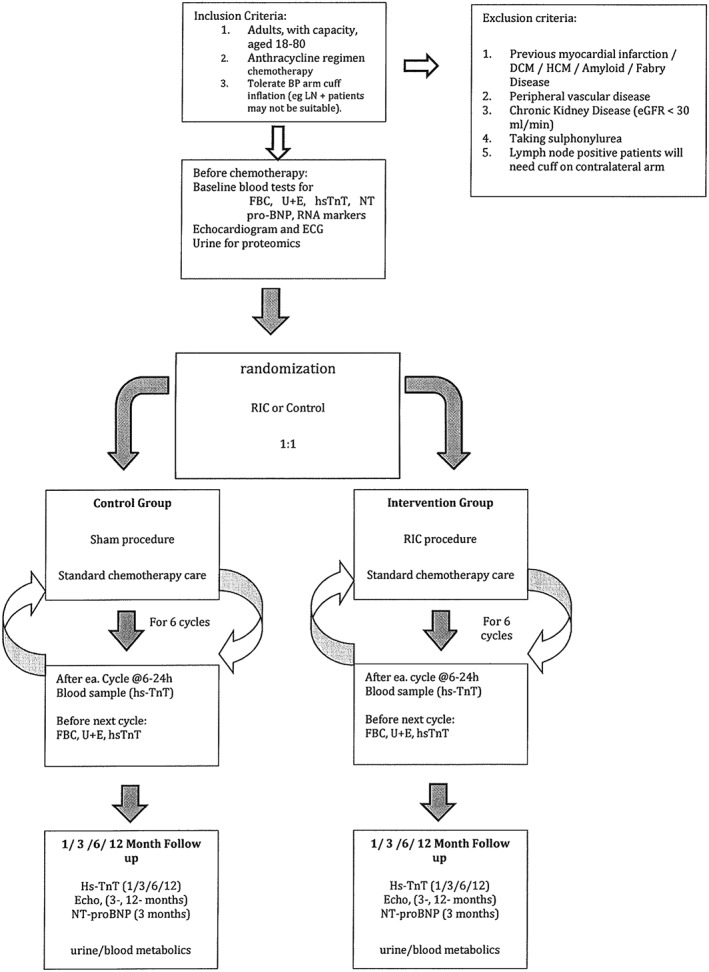
Study flowchart diagram. Abbreviations: BP, blood pressure; DCM, dilated cardiomyopathy; ECG, electrocardiogram; eGFR, estimated glomerular filtration rate; FBC, full blood count; HCM, hypertrophic cardiomyopathy; hsTnT, high‐sensitivity troponin‐T; LN, lymph node; NT pro‐BNP, N‐terminal pro‐brain natriuretic peptide; RIC, remote ischemic conditioning; U + E, urea and electrolytes.

The ERIC‐ONC study will be a single‐center, blinded, randomized, sham‐controlled trial. The ERIC‐ONC study has been reviewed and approved with a favorable opinion from the United Kingdom National Research Ethics Service (NRES) (London‐Chelsea Committee NRES REC reference: 15/LO/1116). The ERIC‐ONC study protocol is registered on the public trials database clinicaltrials.gov NCT 02471885 (Figure 2).

### Study Population

The ERIC‐ONC study will be conducted at a single tertiary cancer referral center in London, United Kingdom. Patient inclusion criteria are summarized in Table 2. Participants will be recruited from adult oncology patients newly referred for anthracycline chemotherapy (Eastern Cooperative Oncology Group performance status <3), 18 to 80 years of age, who can provide informed consent (based on Cancer Research United Kingdom data^38^ documenting one‐third of all new cancers were diagnosed in patients 75 years old and older, with the 50‐ to 74‐year‐old age group accounting for 43% of all cancer deaths).

**Table 2:** Study Participant Inclusion and Exclusion Criteria

Inclusion Criteria	Exclusion Criteria
Adult cancer patients ages 18–80 years	Recent myocardial infarction in previous 4 weeks^a^
Anthracycline regimen chemotherapy	Previous diagnosis of dilated, hypertrophic, cardiac amyloidosis, or Anderson‐Fabry disease
Able to tolerate upper arm blood pressure inflation	Peripheral vascular disease with claudication on symptomatic or imaging criteria
	Chronic kidney disease (estimated glomerular filtration rate <30 mL/min)
	Taking sulphonylureas
	Lymph node dissection/peripherally inserted central line precluding contralateral arm blood pressure cuff inflation

a Acute myocardial infarction/acute coronary syndrome defined according to the European Society of Cardiology, the American College of Cardiology, the American Heart Association, and the World Heart Federation guidelines as detection of a rise of cardiac biomarker (eg, cardiac troponin I/T) with at least 1 value above the 99th centile upper reference limit with at least 1 of the following: symptoms of ischemia, new or presumed new significant ST‐T changes or new left bundle branch block, pathological Q‐waves on electrocardiogram, imaging evidence of loss of viable myocardium or new regional wall motion abnormality, and intracoronary thrombus on angiography.^51^

Patients will be excluded if they have had a recent myocardial infarction (acute coronary syndrome) in the previous 4 weeks, or have a defined cardiac diagnosis that may raise baseline cardiac biomarkers above the reference 99th centile such as dilated cardiomyopathy, hypertrophic cardiomyopathy,^39^ cardiac amyloidosis,^39^, ^40^ or Anderson‐Fabry disease^41^ diagnosed or treated at our tertiary cardiac center. Patients will also be excluded if they have significant peripheral vascular disease defined as upper limb claudication by symptoms or imaging, chronic kidney disease with estimated glomerular filtration rate <30 mL/min/1.73 m^2^, or taking sulfonylureas. Patients unable to tolerate peripheral arm blood pressure cuff inflation due to bilateral lymph node dissection, peripherally inserted central catheter, or significant thrombocytopenia (platelet count <100,000/μL) will be excluded.

### Chemotherapy Regimens

Participants recruited to the study will undergo, as part of their standard cancer treatment, typically 6 cycles of anthracycline‐based chemotherapy occurring at 1 to 3 weekly intervals in the University College London Hospital–Macmillan Cancer Centre. The participant's usual cancer chemotherapy care will only be altered along standard clinical guidelines by their usual oncology team.

### Intervention

The RIC intervention will be delivered as 4 cycles of upper arm blood pressure cuff inflation to 200 mm Hg (or 20 mm greater than systolic blood pressure) for 5 minutes to induce transient noninjurious limb ischemia, followed by cuff deflation for 5 minutes. Participants randomized to the sham control arm will receive 4 cycles of upper arm blood pressure cuff inflation to 10 mm Hg for 5 minutes to simulate treatment, followed by cuff deflation for 5 minutes. Both treatments will last 40 minutes and will be applied before the start of planned chemotherapy. If patients are unable to tolerate all 4 cycles of cuff inflation, they will still be included in the study, as there is evidence that a single cycle of RIC confers cardioprotection.^42^

### Randomization and Blinding

On the day of the first chemotherapy infusion, patients will be randomly allocated in a 1:1 ratio to either the RIC or sham group, using the following minimization factors: diabetes, hypertension, and coronary artery disease to ensure that groups are evenly matched in terms of cardiotoxicity risk factors.^22^, ^43^ Randomization will be performed using MinimPy 0.3 software (http://minimpy.sourceforge.net) on a laptop by an unblinded nurse specialist who will not be involved in data analysis.

### Study Endpoints

The primary outcome measure for the ERIC‐ONC study will be high‐sensitivity troponin‐T (hsTnT) measured at baseline/prechemotherapy cycle, and at 6 to 24 hours after chemotherapy cycles, then at 1, 3, 6, and 12 months follow‐up. Study outcome measures are summarized in Table 1.

Secondary outcome measures will include a composite clinical endpoint for major adverse cardiovascular clinical events (MACCE) at 1, 3, 6, and 12 months follow‐up, defined as myocardial infarction, clinical heart failure requiring admission, life‐threatening cardiac arrhythmia, atrioventricular block requiring pacemaker, or cardiac or cancer death. Participants will be screened at regular study visits for any MACCE leading to admission at their local hospital or the tertiary center, adjudicated by a cardiologist. These data will be entered onto an individual case report form (CRF) and archived electronically onto the Research Electronic Data Capture (REDCap) system.^44^ We intend to perform echocardiograms on a uniform GE E9 ultrasound system (GE Healthcare, Waukesha, WI) to measure ventricular chamber dimensions, ejection fraction, and longitudinal strain at baseline, 3 months, and 12 months using the TomTec Arena (TomTec Imaging Systems, Unterschleissheim, Germany) speckle tracking package at our core lab. Cardiac arrhythmia, at chemotherapy cycle 5/6, will be documented over 14 days (Zio XT ECG patch; CardioLogic, North Yorkshire, United Kingdom), defined as atrial fibrillation/flutter, supraventricular tachycardia, ventricular tachycardia, and atrioventricular block. The oncology team will be notified if clinically significant arrhythmia is documented. N‐terminal pro‐brain natriuretic peptide levels, as a biomarker of raised left atrial pressure and remodeling, will be recorded at baseline and 3 months follow‐up. We will compare in 10 control and 10 RIC participants, changes in mitochondrial DNA, microRNA, and urine proteomics and protein expression markers at baseline and at 3 months.

### Sample Size Calculations

There are no previous studies utilizing RIC to prevent cardiotoxicity in chemotherapy. Most of the existing evidence base for detecting chemotherapy cardiotoxicity has utilized troponin‐I assays. Troponin‐positive thresholds vary between each troponin‐I assay, successive generations, and the lone troponin‐T assay. These disparities complicate power calculations and preclude using the evidence base of troponin‐I cardiotoxicity studies for direct comparison. The wide variation in troponin levels and study sizes is illustrated in Table 3.

**Table 3:** Troponin Assay Levels and Study Characteristics in Chemotherapy Cardiotoxicity Studies

Study	Patient Mix	M:F, Age	Trop+/Sample Size (% Positive)	Baseline Trop+	Tn Values (ng/L)	Troponin Cutoff (ng/L)	Troponin Assay
Cardinale 2000^98^	Advanced cancer with high‐dose chemotherapy^1^	39:165, 45 ± 10 y	65/204 (32%)	0%	1000 ± 400, delta ejection fraction, −18%	500	Stratus II TnI
Cardinale 2002^64^	Breast cancer with high‐dose chemotherapy^2^	211 F, 46 ± 11 y	70/211 (33%)	0%	900 ± 500	500	Stratus II TnI
Cardinale 2004^60^	Advanced cancer with high‐dose chemotherapy	216:487 47 ± 12 y	208/703 (30%)	0 %	E: 160 ± 240, event 1%, 37%, 84% (see below)	80	Stratus CS TnI
Sandri 2003^99^	Advanced cancer with high‐dose chemotherapy	42:137, 47 ± 11 y	(32%) delta ejection fraction 18%	1%	Tn + 630 ± 540 (80–1980), Tn neg = 39 ± 19	80	Stratus II CS TnI
Auner 2003^59^	Hematological adults	32:46, 58 y	78 (15%), delta ejection fraction >10%	0%	Med 40 (30–120)	30	Roche ElecIII TnT
Lipshultz 2004^28^	All children, Dox v, Dex + Dexraz, RCT	120:86, 7.4 y	55/158 (35%)	12/119 (10%)	Tn + 50%,Tn++ 32%; Dex 21%, 10%	10 + 25++	Roche Elecsys TnT
Kilickap 2005^61^	Advanced hematological cancer with high‐dose chemotherapy	20:21, 44 y	(34%)	N = 1 (16 ng/L)		10, ?100, error in article	Roche ElecIII TnT
Haney 2013^100^	Breast cancer	22 F	41% (9/22)	N/A	Peak 60 ng/L, cycle 6: 50% Tn+	Tn+ >12 ng/L; TnT+ 22, samples 91	Roche TnT
Katsurada 2014^49^	Breast cancer, anthracycline + herceptin	19 F only, age N/A	N = 19 hsTnT values	N/A	11 ± 7.8, 4 ± 1.4	14	Roche hs‐TnT

Abbreviations: Dex, Dexraz: Dexrazoxane; Dox: Doxorubicin; E: Early; hsTnT: high‐sensitivity Troponin T; Med: Median; RCT: Randomized Controlled Trial; Tn: Troponin; TnT: Troponin T.

This table illustrates the wide variation in troponin‐I and troponin‐T assays, troponin values, and study sizes. Although not directly comparable, we have converted the levels to nanogram/liter (ng/L) here for simplicity. Peak troponin values in high‐dose chemotherapy studies reached 1980 ng/L. Peak values in low‐dose studies reached 11–120 ng/L. Study sizes ranged from 19–703 participants. Studies routinely classified 30%–40% of patients as “troponin positive” across various generations of troponin assays with differing cutoff values. We formulated several different power calculations to estimate sample size for the study. The calculations were broadly grouped into 2 different models depending on whether we treated the primary outcome measure for troponin levels as a categorical variable (a proportion of patients had a predefined troponin‐positive rise) or as a continuous variable (a difference in troponin rise). If we treated troponin rise as a categorical (dichotomous) variable, whereby we stratified patients into early and late troponin‐positive versus troponin‐negative responses based on Cardinale et al,^60^ a similar study would require either 42 or 586 patients, depending on whether the cardioprotective effect prevented all early and late troponin‐positive events or only early troponin positive events, respectively. Alternatively, treating troponin as a continuous variable and using Cardinale et al^60^ and troponin levels stated in Table 3, the study would require 166 or 630 patients. Using data from Sandri et al 2003,^99^ a study would require between 28 and 190 participants, based on a troponin difference of −590 or −220 ng/L, respectively. Thus, our sample size of n = 128 total study participants falls in the middle range of our calculations for effect size and feasibility.

Power calculations for this pilot study were based on serial troponin measurements treated as continuous outcome variables with a predicted difference. Previous RIC studies in the literature documented a cardioprotective effect of reduced troponin release, ranging from −18 to −62%.^45^, ^46^, ^47^, ^48^ We therefore hypothesized a predicted treatment effect of −35% with 80% power at the 5% significance level, and we calculated a sample size of 128 participants (n = 64 in each arm of the study). This calculation was based on the only hsTnT cardiotoxicity study to date by Katsurada et al^49^ in breast cancer chemotherapy patients with and without echocardiographically defined cardiotoxicity (using peak hsTnT values as parameters to characterize the troponin curve, with control [group 1] hsTnT = 11 ng/L, standard deviation 7.8 ng/L, and RIC intervention [group 2], anticipated −35% difference to 7.15 ng/L, on 1:1 ratio, with an α = 0.05, power = 80%).

### Statistical Analysis

The effect of RIC treatment on hsTnT levels at baseline, at 6 to 24 hours and prior to each chemotherapy cycles, and at 3, 6, and 12 months, will be recorded a continuous variable. Serial data at time points will be compared using a repeated measures mixed effects model. We will analyze missing data by intention to treat analysis and multiple imputation in consultation with a statistician, depending on whether the data were missing at random or not, respectively. Categorical data will be analyzed using Fisher exact test or χ^2^ test if frequency >5. We will use Cox proportional hazards regression to calculate hazard ratios, and the Kaplan‐Meier function to visualize the cumulative incidence for MACCE. Comparisons will be deemed statistically significant with a *P* < 0.05.

### Data Management, Governance, and Funding

The ERIC‐ONC trial is funded under the National Institute of Health Research Biomedical Research Centre and sponsored by University College London. Data will be collected on a CRF and managed using REDCap electronic data capture tools. REDCap^44^ is a secure, Web‐based application designed to support validated and audited data capture for research studies. An independent data monitoring committee will be convened to monitor the progress and safety of the study.

## Discussion

There is no universally accepted definition of cardiotoxicity. Historically, cardiotoxicity has been characterized along clinical grounds (eg, overt heart failure) and more recently by noninvasive imaging based on ejection fraction (ie, either as a symptomatic 5% fall or an asymptomatic 10% fall in ejection fraction to less than normal values).^50^ Current consensus guidelines suggest that biomarker rise may be more sensitive in detecting early asymptomatic cardiotoxicity to guide imaging assessment.^50^

In addition to its central role in the universal definition of ACS^51^ and prognosis,^52^ troponin predicts outcomes in other forms of heart disease,^53^, ^54^, ^55^, ^56^, ^57^, ^58^ as well as anthracycline‐induced cardiotoxicity.^59^, ^60^, ^61^ The evidence base shows that 30% to 40% of anthracycline patients demonstrate a troponin‐positive trend during treatment, but these historical data fluctuate according to troponin assay. Peak troponin‐I/T values, though not directly comparable, range from 11 to 120 ng/L, and 160 to more than 1000 ng/L, in low‐dose and high‐dose anthracycline regimens, respectively (Table 3). Myocardial injury and subsequent troponin release rises progressively with cumulative anthracycline dose.^61^ There is a single manufacturer of the hsTnT assay (Elecsys; F. Hoffmann‐La Roche, Basel, Switzerland). The current generation Roche hs‐TnT assay for the ERIC‐ONC study can be directly compared across all centers utilizing the sole hs‐TnT assay, enabling generalization of results and facilitating comparison in future studies. This quantitative 1‐step troponin‐T enzyme immunoassay is specified with a 10% coefficient of variation upper limit of normal set at <14 ng/L, limit of detection = 5 ng/L, measurement range 3 to 10 000 ng/L.

The most widely used imaging measurement of cardiotoxicity is ejection fraction, either by echocardiogram or multigated acquisition scan, but this measurement has important limitations in terms of precision and repeatability.^62^ Furthermore, morphological studies have shown no linear association between biopsy findings and ejection fraction.^63^ More recent work has demonstrated biomarker evidence of myocardial injury even after the first cycle of anthracycline therapy^60^, ^64^ before a fall in ejection fraction, implying there may be no safe threshold below which detectable myocardial injury does not occur. Participants will undergo clinical and research echocardiograms according to clinical and study protocols. Imaging evidence of subclinical cardiotoxicity, defined as an asymptomatic fall in left ventricular ejection fraction of ≥10%, will be discussed with oncology for initiation of angiotensin‐converting enzyme inhibitor and/or β‐blocker therapy.^65^

Cardiac arrhythmia is common (in excess of 12%) in cancer patients and affects morbidity and mortality,^66^, ^67^ but prospective studies have been relatively small and monitored for short durations.^68^, ^69^ We will monitor prospectively in all 128 participants the incidence of cardiac arrhythmia. Participants will wear an electrocardiogram patch after penultimate cycle 5 of chemotherapy treatment for 14 days to span the period when we expect RIC to confer antiarrhythmic protection^43^, ^69^, ^70^ from the cumulative effect of anthracyclines,^60^, ^61^, ^70^ consistent with a putative role for mitochondrial dysfunction in arrhythmogenesis.^71^

Pharmacological treatments to reduce myocyte damage during chemotherapy include antioxidants, iron chelation, and standard heart failure medications. Antioxidant strategies have proved disappointing^72^, ^73^; therapies such as L‐carnitine, co‐enzyme Q10, N‐acetylcysteine, and phenethylamines have proved disappointing in children and equivocal in adults,^74^ possibly because antioxidants exert their effect after free radical formation. Renin‐angiotensin‐system inhibition,^75^ statin therapy,^76^ and β‐blockers^77^, ^78^ have shown promise in adult cardioprotection,^79^, ^80^ but their universal adoption is constrained by interactions with renal function, transaminitis/myositis, and hemodynamic side effects, respectively. Dexrazoxane is 1 of only 2 iron chelators (along with deferiprone)^27^ that demonstrated cardioprotective effects^28^, ^29^ by limiting cardiac troponin rise in pediatric and adult hematological patients. However, dexrazoxane may cause myelosuppression, and its regulatory license^26^ is limited to cancer patients embarking on extended courses of anthracycline chemotherapy, rather than initial chemotherapy treatment. Furthermore, the incidence of secondary malignancy following dexrazoxane remains highly controversial.^81^, ^82^, ^83^ Thus, to date, pharmacological interventions have demonstrated variable success and their wider use limited by licensing constraints or side effect profiles.

The phenomenon of ischemic conditioning as a form of cardioprotection against ischemia was described by Murry et al in 1986 as activation of endogenous cardioprotective mechanisms to limit myocardial infarct size through repeated cycles of nonlethal ischemia‐reperfusion before an index episode of prolonged ischemic injury.^30^ Ischemic conditioning exerts its cardioprotective effects via ligands such as adenosine, bradykinin, and opioid cell surface receptors acting on downstream signaling cascades involving recruitment of what has been termed the reperfusion injury salvage kinase pathway. This in turn is thought to cause inhibition of MPTP opening and inhibition of reactive oxidation species ROS.^84^ Yellon's group demonstrated that mechanical RIC with a blood pressure cuff^85^, ^86^ could also confer cardioprotection via a noninvasive, nonpharmacological technique.^45^, ^46^

As a nonpharmacological treatment in the setting of elective coronary artery bypass graft surgery, RIC reduced myocardial injury by decreasing troponin rise by 18% to 43%.^45^, ^46^, ^48^ RIC also decreased troponin rise after primary percutaneous coronary intervention (PCI) by 50% to 63%,^47^, ^87^, ^88^ improved myocardial salvage by 26%,^89^ and reduced myocardial infarction size by 27%.^90^ RIC was associated with decreased frequency of atrial fibrillation by more than 50% in coronary artery bypass grafting (CABG)^45^ and abolished it in a small series of aortic valve replacement patients.^91^ RIC decreased the incidence and extent of acute kidney injury by 30% in the setting of elective high‐risk cardiac surgery.^92^ It improved medium‐term clinical outcomes by reducing (MACCE after CABG at 1.5 years,^48^ and in primary PCI by more than 40% at a median follow‐up of 3.8 to 4.3 years.^87^, ^93^ RIC appears to exert a neutral effect on outcomes in CABG surgery (the ERICCA study [Effect of Remote Ischaemic Preconditioning on Clinical Outcomes in CABG Surgery]; clinicaltrials.gov identifier NCT01247545),^94^, ^95^ potentially due to the use of intraoperative propofol.^95^ Long‐term outcomes in PCI are being investigated (ERIC‐PPCI study [Effect of Remote Ischaemic Conditioning on Clinical Outcomes in STEMI Patients Undergoing PPCI]; clinicaltrials.gov NCT02342522). Cuff inflation is well‐tolerated with few side effects. Martin‐Gill et al^96^ reported the tolerability of RIC with 83% of 99 air ambulance transport patients tolerating 3 or 4 cycles of RIC over 25 to 35 minutes, with 53% reporting no pain and only 5% reporting moderate 5 out of 10 discomfort. Similarly, Li et al^97^ reported no adverse effects in 216 patients.

To date, there are few studies applying RIC to organ protection in cancer treatment and no comparable studies investigating RIC cardioprotection in the setting of cancer chemotherapy. Li et al^97^ demonstrated the effectiveness of RIC in attenuating acute lung injury in cancer patients, reducing oxygenation requirements and postoperative stay in lung resection surgery. Thus, RIC confers cardioprotection, renal protection, and lung protection against ischemic injury.

The ERIC‐ONC trial will be the first study to investigate the efficacy of remote ischemic conditioning as a novel form of noninvasive, nonpharmacological cardioprotection against anthracycline chemotherapy. We anticipate our study will establish the effectiveness of RIC to precondition and protect the heart against subclinical biochemical, structural, electrical, and metabolic cardiac injury in the cardio‐oncology setting as a pilot for a future multicenter trial.
